# “Case report”: Whole-exome sequencing reveals compound heterozygous variants in the *EIF2B5* gene in a familial case of vanishing white matter

**DOI:** 10.3389/fgene.2025.1688885

**Published:** 2025-10-31

**Authors:** Sofía Savy, Francisco A. Montes, Carola L. Grosso, Laura E. Laróvere, Silene M. Silvera-Ruiz, Gerardo H. Carro, Guillermo Guelbert, Adriana Becerra, David Morales, Juan P. Nicola

**Affiliations:** ^1^ Departamento de Bioquímica Clínica, Facultad de Ciencias Químicas, Universidad Nacional de Córdoba, Córdoba, Argentina; ^2^ Centro de Investigaciones en Bioquímica Clínica e Inmunología - Consejo Nacional de Investigaciones Científicas y Técnicas (CIBICI-CONICET), Córdoba, Argentina; ^3^ Centro de Estudio de las Metabolopatías Congénitas, Hospital de Niños de la Santísima Trinidad, Córdoba, Argentina; ^4^ Cátedra de Clínica Pediátrica, Facultad de Ciencias Médicas, Universidad Nacional de Córdoba, Córdoba, Argentina; ^5^ Sección de Enfermedades Metabólicas, Hospital de Niños de la Santísima Trinidad, Córdoba, Argentina; ^6^ Servicio de Neurología, Hospital Nuestra Señora de la Misericordia, Córdoba, Argentina

**Keywords:** leukodystrophy, vanishing white matter (VWM), psychomotor slowing, whole-exome sequencing, eukaryotic translation initiation factor 2B (EIF2B), loss-of-functionEIF2B5 variants

## Abstract

Vanishing white matter (VWM) is a rare autosomal recessive leukodystrophy associated with pathogenic variants in any of the five genes (*EIF2B1-5*) that encode subunits of the eukaryotic translation initiation factor 2B (eIF2B). Here, we present a case of a 26-year-old female patient from a non-consanguineous Amerindian Bolivian family, with clinical and neuroimaging findings suggestive of early-onset VWM, characterized by slowly progressive neurological deterioration in the absence of ovarian disorder. Whole-exome sequencing revealed a novel pair of compound heterozygous variants in the *EIF2B5* gene, confirming the diagnosis of leukodystrophy with VWM and bringing closure to a nearly 20-year diagnostic odyssey. The identified c.318A>T (p.Leu106Phe) and c.1688G>A (p.Arg563Gln) in the *EIF2B5* gene were classified as pathogenic and likely pathogenic, respectively, according to the American College of Medical Genetics and Genomics. Complementary Sanger sequencing revealed that the variants co-segregated with the phenotype in the pedigree, providing strong evidence of autosomal recessive inheritance of the disease, and enabling the molecular diagnosis of two asymptomatic sisters with white matter lesions on neuroimaging. This case underscores the heterogeneous nature of VWM, and emphasizes the relevance of integrating a comprehensive clinical evaluation, brain magnetic resonance imaging, and genetic studies in the diagnosis of leukodystrophies.

## Introduction

Leukodystrophy is a broad term used to describe all inherited genetic disorders of the white matter in the central nervous system, with or without peripheral nervous system involvement (Vanderver et al., 2015). Their differential diagnosis is very broad, and as such, neuroimaging information can be very useful in approaching an early diagnosis (Davies et al., 2023). Among the most prevalent leukodystrophies, vanishing white matter disease (VWM)–also called childhood ataxia with central hypomyelination–is a rare autosomal recessive disease causing a chronic deterioration of the white matter in the brain and spinal cord, leading to progressive neurological deterioration with cerebellar ataxia, usually less prominent spasticity and relatively mild mental decline (Hamilton et al., 2018). While childhood onset is the most common form of the disease, severe forms are apparent at birth, and milder forms may not become evident until adolescence or adulthood. A characteristic clinical hallmark of the disease is its generally uneven progression, marked by periods of relative stability interrupted by episodes of rapid and severe neurological deterioration, often triggered by stresses, such as fever, minor head trauma, acute psychological stress, or infection, which can provoke the onset or worsening of symptoms and may lead to lethargy or coma (Hamilton et al., 2018).

VWM is caused by biallelic loss-of-function variants in any of the five genes (*EIF2B1-5*) that encodes the five subunits (alpha-epsilon) of the eukaryotic translation initiation factor 2B (eIF2B), a guanine nucleotide exchange factor that plays a crucial role in regulating protein synthesis (Leegwater et al., 2001). Impaired eIF2B function triggers improper activation and dysregulation of the integrated stress response rendering glial cells, specifically oligodendrocytes and astrocytes, hypersensitive to physiological cellular stress. As a result, glial cell dysfunction leads to impaired maintenance and repair of myelin, ultimately causing progressive myelin loss and the characteristic white matter degeneration observed in the disease (Wong et al., 2019).

Magnetic resonance imaging findings are diagnostic, showing a diffuse abnormality of the cerebral white matter, beginning in the presymptomatic stage (Takano et al., 2015). Given the overlapping clinical features with other leukodystrophies and the genetic heterogeneity of VWM, accurate molecular diagnosis through comprehensive genetic testing, such as whole-exome sequencing, is essential for guiding clinical management and providing accurate genetic counseling. Here, we present a case of a 26-year-old female patient with clinical and imaging findings suggestive of early-onset VWM presenting a slowly progressive neurological deterioration without ovarian disorder. Whole-exome sequencing testing revealed a novel pair of compound heterozygous variants in the *EIF2B5* gene, confirming the diagnosis of leukodystrophy with VWM and bringing an end to a nearly 20-year diagnostic odyssey.

## Materials and methods

### Ethics statement

This study was approved by the Comité Institucional de Ética de la Investigación en Salud (CIEIS) del Niño y del Adulto Polo Hospitalario, Ministerio de Salud de la Provincia de Córdoba, Argentina. Informed written consent was obtained from all patients enrolled in the study.

### Genomic DNA isolation

Genomic DNA was isolated from ethylenediaminetetraacetic acid-anticoagulated whole blood using the standard cetyltrimethylammonium bromide-based method (Geysels et al., 2022).

### Whole-exome sequencing and data processing

Whole-exome sequencing was performed by Macrogen (Seoul, South Korea) using an Illumina platform with 150-bp paired-end reads. Exome capture and library preparation were conducted using the Twist Human Core Exome kit (Twist Bioscience, San Francisco, CA). Data analysis was conducted following the recommendations of the Broad Institute Genome Analysis Toolkit for preprocessing, variant calling, and refinement, as previously reported (Carro et al., 2024). Raw reads were mapped to the reference human genome (GRCh38) using the BWA-MEM algorithm of the Burrows-Wheeler Aligner software and visualized using the Integrative Genomics Viewer v.1.4.2. Duplicates were removed using Picard. Variant calling was performed using HaplotypeCaller, and variant annotation was carried out using ANNOVAR, resulting in the generation of annotated variant call format (VCF) file.

### Genetic data analysis

The variants identified by whole-exome sequencing were ranked using the online tool Franklin (https://franklin.genoox.com/). Variants with a minor allele frequency higher than 0.1% in the Genome Aggregation Database version 4.1.0 (https://gnomad.broadinstitute.org/) were excluded. Phenotypic filtering based on the Human Phenotype Ontology (HPO) criteria related to the patients’ clinical features, including leukodystrophy (HP:0002415), seizure (HP:0001250), and psychomotor deterioration (HP:0002361), was applied.

The meta-predictor Rare Exome Variant Ensemble Learner (REVEL) was used to predict the pathogenicity of rare missense variants, with benignity (BP4) and pathogenicity (PP3) evidence strength annotated per variant using the recommended thresholds (≤0.290 and ≥0.644, respectively) (Pejaver et al., 2022). Gene function and possible associated diseases were evaluated using the databases GeneCards (https://www.genecards.org/) and OMIM (https://www.omim.org/) in addition to relevant literature research on PubMed (https://pubmed.ncbi.nlm.nih.gov/).

Variants were named according to the recommendations of the Human Genome Variation Society (https://hgvs-nomenclature.org). Variant interpretation was determined according to guidelines formulated by the American College of Medical Genetics and Genomics (ACMG) in a quantitative Bayesian framework and summarized into a point-based system (Tavtigian et al., 2020), including ClinGen’s Criteria-Specific Recommendations.

### Sanger sequencing

A standard polymerase chain reaction procedure was used to amplify selected *EIF2B5* gene exons (Martin et al., 2019). Gene-specific oligonucleotide sets were as follows: exon 2: 5′-TGC​CAC​AGG​TGT​ACA​GGA​AA (forward) and 5′-TTC​CCC​TCT​CAC​ATT​TCC​CC (reverse), and exon 12: 5′-TCT​GCC​TGG​ATC​AAC​TAG​CC (forward) and 5′-GAG​AAC​AGG​GAG​GGG​CTG (reverse). The PCR products were resolved by electrophoresis on 2% agarose gels containing ethidium bromide. The resulting bands were then purified using a QIAquick PCR Purification Kit (Qiagen, Hilden, Germany) as previously reported (Bernal Barquero et al., 2022). The nucleotide sequence of the PCR products was determined by Sanger sequencing by capillary electrophoresis in an ABI 3500xL Genetic Analyzer (Applied Biosystems, Foster City, CA). Chromatograms were analyzed using Benchling (https://www.benchling.com/).

## Results

We present the case of a female patient of Amerindian Bolivian origin who was diagnosed with epilepsy at the age of eight. Her first seizure occurred at the age of two, approximately 48 h after a mild traumatic brain injury. Brain magnetic resonance imaging at that time suggested a white matter disorder consistent with leukodystrophy, although the imaging is no longer available in the medical record. Initial metabolic studies, including plasma long-chain fatty acids and arylsulfatase A enzyme activity, were within the reference intervals.

**FIGURE 1:**
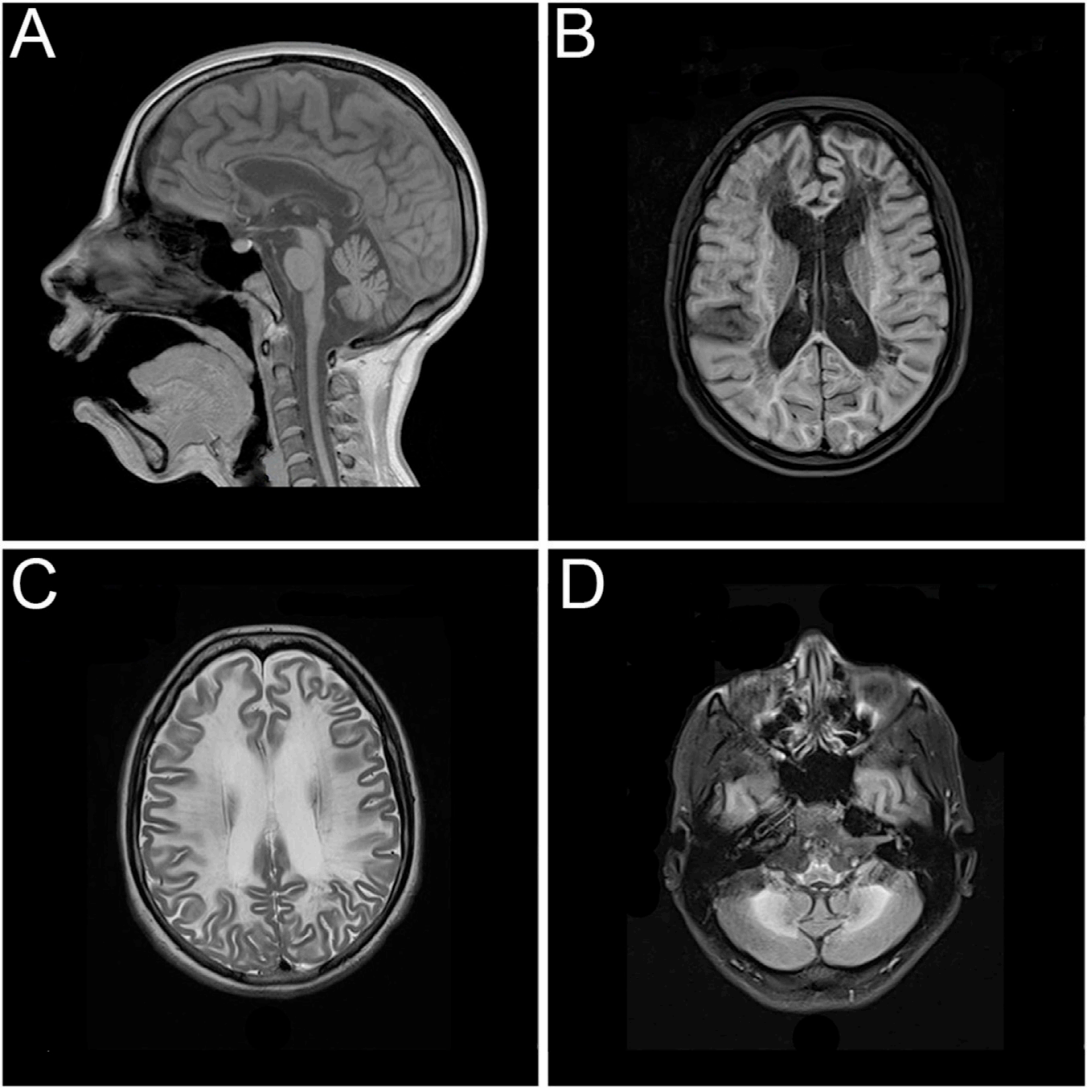
Magnetic resonance imaging showing extensive and symmetrical changes in bilateral cerebral hemisphere demyelination with glial hyperplasia involving the corpus callosum. **(A)** Mid-sagittal T1-weighted image. **(B)** Axial T1-weighted image. **(C)** Axial T2-weighted image. **(D)** Axial T2-weighted image.

After attending to several medical institutions without receiving a definitive diagnosis and enduring a long diagnostic odyssey, the patient returned for an evaluation at the age of 26 due to clinical progression, presenting with marked psychomotor slowing and gait disturbance. A follow-up brain magnetic resonance imaging showed increased depth of the cerebellar sulci, diffuse thinning of the corpus callosum, enlargement of the ventricular system associated with deeper sulci and fissures, and marked hyperintensity of the periventricular, paraventricular, and frontoparietotemporooccipital subcortical white matter. Extensive lesions with a sequelae-like appearance, with areas of diffuse leukomalacia of the centrum ovale and periventricular white matter bilaterally and symmetrically, even extending to the temporal, anterior, and basal regions. Deposition of paramagnetic metabolites were observed in the caudate nucleus, putamen, thalamus, subthalamic region, and dentate nuclei of the cerebellar, and marked hyposignal on the magnetic susceptibility sequence (Figure 1). The patient provided an electroencephalogram conducted at another institution performed at age 24, which showed globally disorganized and slowed background activity without evidence of epileptiform abnormalities. Further metabolic investigations—including analyses of urinary organic acids, plasma and urinary amino acids, urinary purines and pyrimidines, plasma homocysteine and very long-chain fatty acids, plasma and urinary creatine metabolites, and plasma arylsulfatase A enzyme activity—were all within normal reference ranges. Enzymatic assays ruled out neuronal ceroid lipofuscinosis types I and II.

**FIGURE 2:**
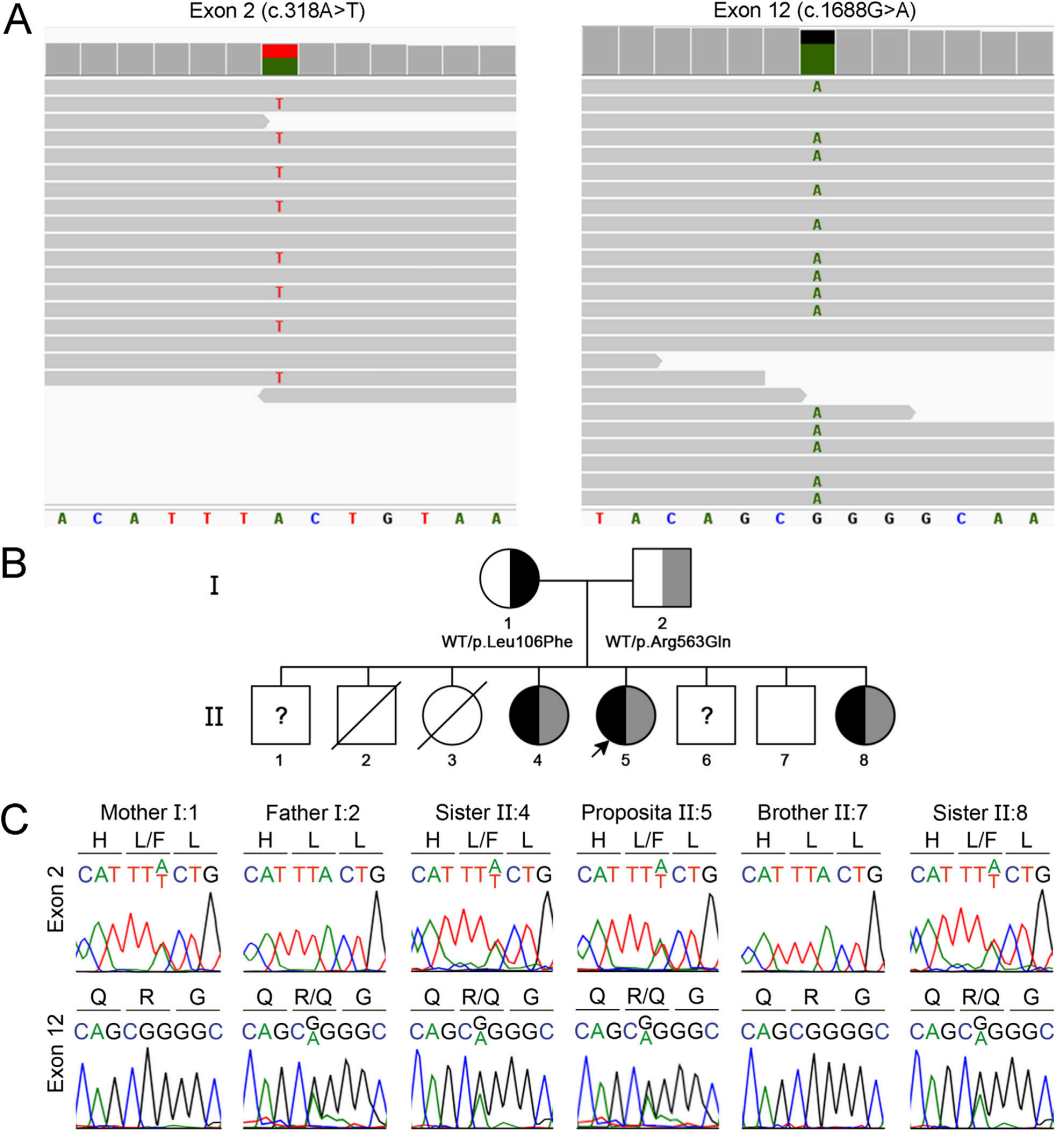
Identification of compound heterozygous missense *EIF2B5* gene variants causing VWM. **(A)** Visualization with Integrative Genomics Viewer of whole exome sequencing reads aligned with the reference human genome (GRCh38) showing *EIF2B5* gene variants c.318A>T and c.1688G>A. **(B)** Segregation of the *EIF2B5* gene variants within the proband’s family. **(C)** Sanger sequencing chromatogram showing a 9-bp fragment of EIF3B5 exon 2 (nucleotides 313 to 321) and exon 12 (nucleotides 1,684 to 1,692). Amino acids are indicated using the one-letter code. The proband and sisters II.4 and II.8 are compound heterozygous for the variants c.318A>T, p.Leu106Phe and c.1688G>A, p.Arg563Gln. The mother carries the variant c.318A>T and the father carries the variant c.1688G>A. The variants were absent in the healthy brother II.7. The healthy brothers II.1 and 6 were not available for genetic analysis.

Whole-exome sequencing testing was performed based on the clinical and imaging suspicion of leukodystrophy of genetic origin. Two heterozygous missense variants in the *EIF2B5* gene were identified: c.318A>T, p.Leu106Phe, and c.1688G>A, p.Arg563Gln, which are located in exons 2 and 12, respectively (GenBank Reference Sequence NM_003907.3; MANE Select Transcript) (Figure 2A). Pathogenic variants in the *EIF2B5* gene, which encodes the epsilon subunit of the eukaryotic translation initiation factor 2B (eIF2B), are associated with autosomal recessive leukodystrophy with VWM, which is frequently—but not invariably—accompanied by ovarian disorder in females (Ibitoye et al., 2016). In the present case, the patient reported regular menstrual cycles and normal menstrual flow, suggesting preserved ovarian function.

The c.318A>T variant has been reported in the Single Nucleotide Polymorphism database (rs113994048), showing a total allele frequency of 0.000009913 according to The Genome Aggregation Database (PM2_Supporting evidence of pathogenicity), and has been reported in ClinVar as pathogenic (Variation ID: 195203). *In silico* analysis using REVEL predicted the variant p.Leu106Phe as pathogenic, with a score of 0.73 (PP3_Supporting evidence of pathogenicity). Previous reports in the literature have described this variant in both homozygosity and compound heterozygosity in unrelated patients with VWM (PM3_VeryStrong evidence of pathogenicity) (Leegwater et al., 2001; Horzinski et al., 2009; Turon-Vinas et al., 2014; Cohen et al., 2020). Functional studies performed in *Saccharomyces cerevisiae* using the yeast orthologue of *EIF2B5* demonstrated that the Leu106Phe substitution results in the reduction of the expression of the affected protein subunit (Richardson et al., 2004). The c.1688G>A variant has also been reported in the Single Nucleotide Polymorphism database (rs1290134782), showing a total allele frequency of 0.000003718 according to The Genome Aggregation Database (PM2_Supporting evidence of pathogenicity). *In silico* analysis using REVEL predicted the variant p.Arg563Gln as pathogenic, with a score 0.68 (PP3_Supporting evidence of pathogenicity). The variant has been previously identified in compound heterozygosity with the variant c.806G>A, p. Arg269Gln in a patient with VWM (PM3 level of evidence) (Bektas et al., 2018). According to the ACMG guidelines, the c.318A>T variant was classified as pathogenic (PM2_Supporting, PP3_Supporting, PM3_VeryStrong), while the c.1688G>A variant was classified as a variant of uncertain significance (PM2_Supporting, PP3_Supporting, PM3) (Table 1), indicating that there is insufficient evidence to be considered a disease-causing variant.

**TABLE 1:** *EIF2B5* gene variants identified using whole-exome sequencing.

Variant	Effect	Total allele frequency	REVEL	ACMG criteria	ACMG classification
c.318A>T, p.Leu106Phe	Missense	0.000009913	0.73	PM2_Supporting, PP3, PM3_VeryStrong PP1_Moderate	Pathogenic
c.1688G>A, p.Arg563Gln	Missense	0.000003718	0.68	PM2_Supporting, PP3, PM3_Strong, PP1_Moderate	Likely pathogenic

Family history was remarkable for multiple affected siblings (Figure 2B). The proband (II.5) was a full-term female infant born as the fifth of eight siblings to non-consanguineous and healthy Amerindian Bolivian parents. The patient has three healthy brothers (II.1, II.6, and II.7) and two asymptomatic sisters (II.4 and II.8), who are 29 and 18 years old. The sisters showed white matter lesions on neuroimaging (Supplementary Figure S1) and reported regular menstrual cycles, which suggest a slower rate of disease progression. Neither the patient nor her asymptomatic sisters underwent cognitive or neuropsychiatric testing. Two older siblings, one male (II.2) and one female (II.3), died at 2 days and 2 years of age, respectively, without receiving a definitive diagnosis. Sanger sequencing-based segregation analysis of the variants identified using whole-exome sequencing was performed in the family. The *EIF2B5* gene variants were further confirmed in the patient (Figures 2B,C). Consistent with the recessive nature of the disease, analysis of the parents showed that the mother is heterozygous for the c.318A>T variant, and the father for the c.1688G>A variant. Significantly, the two affected sisters (II.4 and II.8) carry the same compound heterozygous genotype of the patient (Figures 2B,C), while the variants were absent in the younger healthy brother (II.7). The other healthy brothers (II.1 and II.6) were not included in the study. Therefore, segregation analysis confirms that the patient and the two affected sisters carry the variants c.318A>T and c.1688G>A in a compound heterozygous state, supporting the application for in trans criterion PM3_Strong for the c.1688G>A variant, and the criterion PP1_Moderate due to cosegregation of the variants in multiple affected family members (proband II.5 and her sisters II.4 and 8) (Figure 2B). In addition, segregation evidence supports the re-classification of the c.1688G>A variant, which was initially classified as a variant of uncertain significance, as likely pathogenic (PM2_Supporting, PP3_Supporting, PM3_Strong, PP1_Moderate) (Table 1). Significantly, the re-classification improves the confidence of the genetic diagnosis, having a direct impact on clinical decision-making, and informed genetic confidence for potential carriers.

**FIGURE 3:**
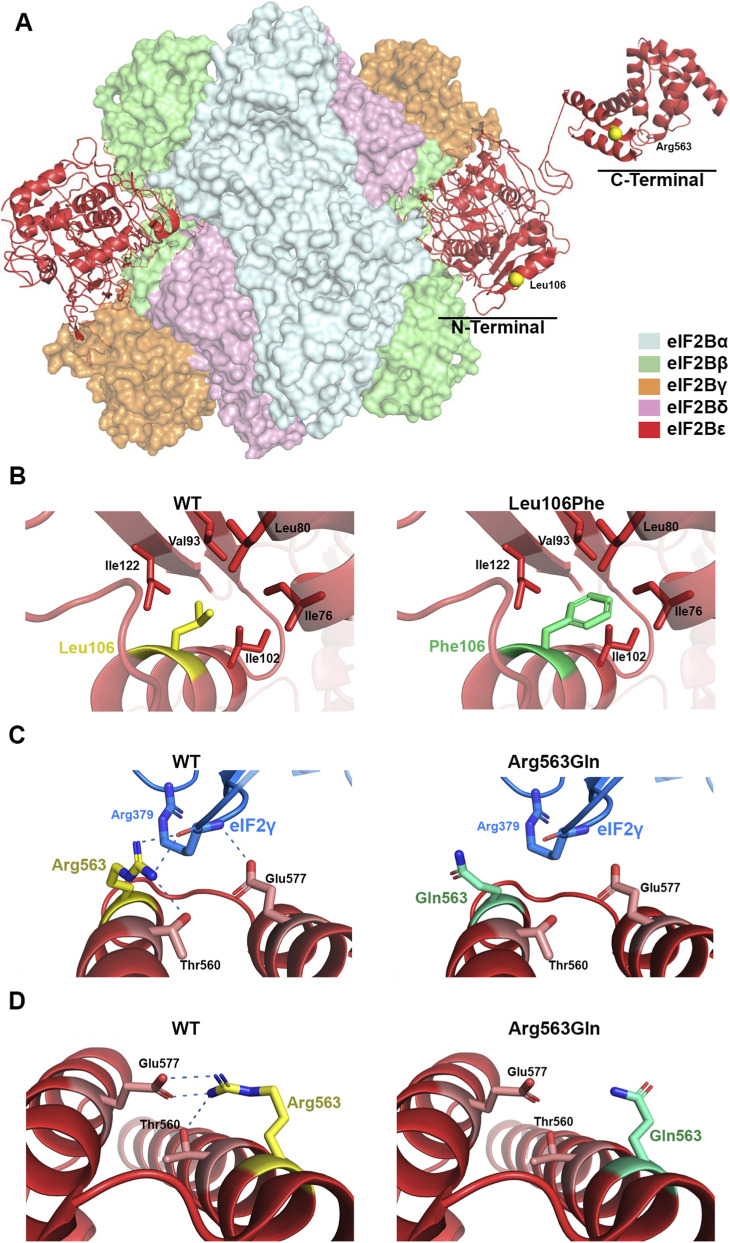
Structural analysis of pathogenic variants in the eIF2Bε protein. **(A)** Overall structure of the human eIF2B decameric complex (PDB ID: 6K71), highlighting in yellow the positions of the residues Leu106 and Arg563 in the eIF2Bε subunit. **(B,C)** Close-up views of the eIF2Bε subunit in complex with eIF2 (PDB ID: 6K71), comparing the wild-type residues with the modeled pathogenic variants Leu106Phe and Arg563Gln. **(D)** Close-up of the catalytic domain (helices I and II) in the C-terminal region of eIF2Bε (PDB ID: 3JUI), showing the wild-type residue and the modeled Arg563Gln variant. Dotted lines indicate the predicted ionic and polar interactions of the Arg563 guanidinium group. Structural representations were generated using Visual Molecular Dynamics (http://www.ks.uiuc.edu/Research/vmd/).

The eIF2B hetero-decameric complex consists of α_2_ (βδ)_2_ hexameric regulatory subcomplex located at the center, with (γε)_2_ heterodimeric catalytic subcomplexes bound on opposite peripheral sides (Figure 3A). The human epsilon eIF2B subunit (eIF2Bε) has an amino-terminal region involved in the interaction with the regulatory subcomplex and a carboxy-terminal region (residues 527–726) containing the catalytic domain (residues 527-588) for eukaryotic translation initiation factor 2 (eIF2)-dedicated guanine nucleotide exchange factor activity. Based on eIF2B structure, the Leu106Phe and Arg563Gln variant are located in the amino-terminal and catalytic domain, respectively (Figure 3A). As previously described (Slynko et al., 2021), the residue Leu106 faces a small hydrophobic cavity formed by residues Ile76, 102, and 122, Leu80, and Val93 (Figure 3B). Thus, a bulky hydrophobic residue at this position—such as Phe—could promote favorable hydrophobic interactions with surrounding residues but might also cause steric restrictions, altering the folding in this region required to accommodate the larger side chain. In line, FoldX calculations indicated that the Leu106Phe variant exerts a substantial destabilizing effect on eIF2Bε protein stability (ΔΔG = 2.71 ± 1.29 kcal mol^-1^, n = 20; 95% CI: 2.10–3.31 kcal mol^-1^). On the other hand, the residue Arg563 is located in a polar region at the amino-terminal of catalytic domain containing catalytically important residues including the critical residue Glu577 (Mohammad-Qureshi et al., 2008), where it seems to play a dual role during the structural rearrangement of the eIF2B complex upon binding either to eIF2 or to the inhibitory phosphorylated eIF2 (Ito et al., 2023). In the productive complex, in which eIF2B catalyzes nucleotide exchange on eIF2, Arg563 appears to participate in direct interactions with eIF2γ similarly to the critical catalytic residue Glu577 (Figure 3C). By contrast, in the non-productive complex, in which the nucleotide exchange reaction is restricted, the catalytic domain of eIF2Bε does not engage with eIF2γ and remains flexibly positioned, Arg563 likely forms ionic interactions with Glu577 and polar interactions with Thr560. These interactions are lost in the Arg563Gln variant (Figure 3D), thereby compromising the correct folding of the catalytic domain. Consistent with this, FoldX calculations predicted that Arg563Gln exerts a moderate destabilizing effect on eIF2Bε protein stability (ΔΔG = 0.89 ± 0.18 kcal mol^-1^, n = 5; 95% CI: 0.67–1.12 kcal mol^-1^). Unfortunately, due to technical limitations, we were not able to perform functional assays to assess the impact of the Arg563Gln variant on eIF2Bε protein expression or function, and thus could not provide PS3-level evidence to support the pathogenicity of the variant. The functional importance of residues Leu106 and Arg563 is further supported by multiple sequence alignment, in which ClustalW2 showed that both missense variants affect highly conserved positions (data not shown).

## Discussion

VWM is a progressive leukodystrophy characterized by loss of cerebral white matter, often triggered by stress-related events. The disease is caused by biallelic loss-of-function variants in any of the five housekeeping *EIF2B* genes, which encode the subunits of the decameric protein complex eukaryotic translation initiation factor 2B (eIF2B), which plays a critical role in eukaryotic cells as a key regulator of protein synthesis initiation (Leegwater et al., 2001). Missense *EIF2B5* gene variants are the most frequent observed genetic defect in VWM (Deng et al., 2021), and are thought to impair the function of the eIF2B complex by disrupting its guanine nucleotide exchange activity, which is essential for recycling eIF2-GDP to eIF2-GTP during protein translation initiation. This activity becomes particularly critical under cellular stress conditions, when the phosphorylation of eIF2α inhibits global protein synthesis to promote stress recovery. Dysfunctional eIF2B complex impairs the cellular adaptive response to stress, resulting in increased vulnerability—particularly in oligodendrocytes and astrocytes, which play essential roles in myelin maintenance. Consequently, glial cell dysfunction disrupts the maintenance and repair of myelin, ultimately leading to progressive myelin loss and the characteristic white matter degeneration observed in VWM (Wong et al., 2019).

Here, we present a case of a 26-year-old female patient with clinical and imaging findings suggestive of early-onset VWM, characterized by slowly progressive neurological deterioration and no evidence of ovarian disorder to date, although the future progression of the disease in this tissue remains unknown. The patient was diagnosed with epilepsy at the age of eight, showing her first seizure at the age of two after a mild traumatic brain injury. Brain magnetic resonance imaging suggested a white matter disorder consistent with leukodystrophy. However, the absence of additional clinical manifestations beyond epilepsy, together with the unavailability of genetic testing at that time, precluded a definitive diagnosis, while vanishing white matter disease and mitochondrial disorders were considered as presumptive diagnoses. During the following years, particularly as psychomotor slowing and gait disturbance increasingly impacted her quality of life, the patient sought care at multiple medical institutions without receiving a definitive diagnosis. This experience reflects the prolonged and challenging diagnostic odyssey commonly associated with genetic leukodystrophies (Parikh et al., 2015). Despite extensive clinical and biochemical evaluations and neuroimaging studies, the underlying etiology remained elusive for nearly 20 years, delaying appropriate medical management and contributing to prolonged diagnostic uncertainty. The diagnosis of leukodystrophies is often delayed due to their rarity of the disease and broad spectrum of nonspecific symptoms, which in adults are frequently mistaken for other neurological disorders. Our experience underscores the urgent need to educate clinicians about these conditions to improve early recognition and facilitate timely referrals. Raising awareness, together with the implementation of electronic health records to ensure continuity of care and accessibility of relevant medical information across the healthcare system, could help prevent similar delays in the future.

Comprehensive clinical evaluation, brain magnetic resonance imaging, and genetic studies are key components in the early diagnosis of leukodystrophies (Deng et al., 2021). Although neuroimaging findings are diagnostic in most patients, definitive diagnosis relies on the identification of pathogenic variants in disease-associated genes. The clinical application of next-generation sequencing technologies has significantly improved the molecular diagnosis of leukodystrophies, resulting in shorter time to diagnosis, higher diagnostic yields, and improved cost-effectiveness during the etiological evaluation phase (van der Knaap and Bugiani, 2017). While no cure currently exists for most heritable white matter disorders, ongoing research into potential therapeutic targets offers hope for future interventions (van der Knaap et al., 2022). Early diagnosis remains critical, not only for providing accurate genetic counseling but also for implementing symptomatic treatments that can substantially reduce disease burden and improve quality of life.

A definitive diagnosis often requires specific genetic testing, which may not be widely available or may be costly, further delaying the diagnostic process. Here, whole-exome sequencing was conducted based on the clinical and imaging suspicion of leukodystrophy revealing a novel pair of compound heterozygous missense variants (p.Leu106Phe and p.Arg563Gln) in the *EIF2B5* gene, supporting the diagnosis of VWM in the proband. Notably, whole-exome sequencing in patients with persistent, unexplained white matter abnormalities has been shown to achieved a diagnostic yield of 42% (Vanderver et al., 2016). Nevertheless, the technique has recognized limitations, including reduced capacity to detect structural and non-coding variants, so a normal result does not exclude a genetic etiology of symptoms. Complementary Sanger sequencing confirmed the compound heterozygous state, with each parent carrying one of the variants, and enabled the diagnosis in two asymtomatic sisters (II.4 and II.8) presenting white matter abnormalities on neuroimaging, and preserved ovarian function. These variants co-segregated with the phenotype in the pedigree, providing strong evidence of autosomal recessive inheritance and supporting the pathogenic role of the identified alterations. The identified p.Leu106Phe and p. Arg563Gln variants were classified as pathogenic and likely pathogenic, respectively, according to ACMG guidelines. While the p.Leu106Phe variant was frequently identified in homozygosity or compound heterozygosity in patients with VWM, the p.Arg563Gln variant has been reported once in compound heterozygosity with the variant p.Arg269Gln (Bektas et al., 2018). Previous studies that compiled cases of ovarioleukodystrophy due to *EIF2B5* variants have shown that the p.Arg113His variant is the most frequent, whether it is in a homozygous or compound heterozygous state (Labauge et al., 2009; Kong et al., 2022). The variants p.Leu106Phe and p.Arg563Gln were not yet reported in patients with ovarioleukodystrophy.

The severity of VWM varies widely, and the factors underlying this broad phenotypic spectrum—even within the same family, as observed in this report—remain poorly understood. A comprehensive evaluation of the natural course of VWM revealed that symptoms developed following exposure to stress-triggering factors—most commonly fever and head trauma—in approximately 70% of patients, while nearly 90% experienced disease exacerbation after successive exposures to such triggers (Hamilton et al., 2018). In contrast to her asymptomatic sisters, the proband developed neurological symptoms after a mild traumatic brain injury in childhood, which may have contributed to the phenotypic heterogeneity observed within the family. To date, genotype–phenotype correlations remain incompletely understood, yet they are crucial for elucidating disease pathogenesis, predicting prognosis, and guiding genetic counseling. Previous studies have identified age at onset as the only independent clinical predictor of disease severity (Hamilton et al., 2018). The nature of the mutation might affect the activity of the EIF2B complex. Indeed, *in silico* protein modeling analysis revealed that the influence of some missense variants in *EIF2B5* are associated with severe phenotypes due to their substantial predicted impact on eIF2B structure, while others appear to have minimal functional consequences and correlate with milder disease severity presentations. Particularly, the p.Leu106Phe variant was predicted to have a severe effect on protein structure and, in the homozygous state, was associated with a severe phenotype and early disease onset (Slynko et al., 2021). However, recent findings suggested that genotype-phenotype correlation requires consideration of the combined effects of biallelic variants, as in cases of compound heterozygosity involving two distinct missense mutations, both alleles contribute to determining the clinical phenotype (Deng et al., 2021). The phenotype of patients carrying compound heterozygous *EIF2B5* variants such as p.Leu106Phe/p.Arg113His or p. Arg563Gln/p.Arg269Gln has been reported to be milder than that observed in patients with homozygous p. Leu106Phe or p.Arg269Gln variants (Horzinski et al., 2009; Turon-Vinas et al., 2014; Bektas et al., 2018; Wang et al., 2016). Based on the natural course of the disease observed in our proband and her affected sisters, we infer that the p.Arg563Gln variant is likely associated with a milder disease presentation, in agreement with structural predictions showing a moderate effect on protein stability.

## Conclusion

These findings underscore the diagnostic value of whole-exome sequencing in rare neurological disorders and emphasize the importance of variant reclassification as new familial or functional evidence becomes available. In this case, the integration of clinical, neuroimaging, and genetic data was essential to establishing a definitive molecular diagnosis. Furthermore, this case illustrates the utility of whole-exome sequencing in complex neurological presentations, particularly leukodystrophies. These conditions are often characterized by overlapping clinical phenotypes, extensive genetic heterogeneity, and a lack of reliable diagnostic biomarkers—factors that frequently contribute to delayed or missed diagnoses.

## Data Availability

The data presented in the study are deposited in the ClinVar repository, accession numbers SCV006586712 and SCV006586713.
