# Supplementary material for: “Case report”: Whole-exome sequencing reveals compound heterozygous variants in the EIF2B5 gene in a familial case of vanishing white matter
Source: Front Genet. 2025 Oct 31;16:1688885. doi: 10.3389/fgene.2025.1688885 (PMC12614830; doi:10.3389/fgene.2025.1688885)
Supplement: Supplementary file 1 [file Table1.docx]

**Supplementary material**


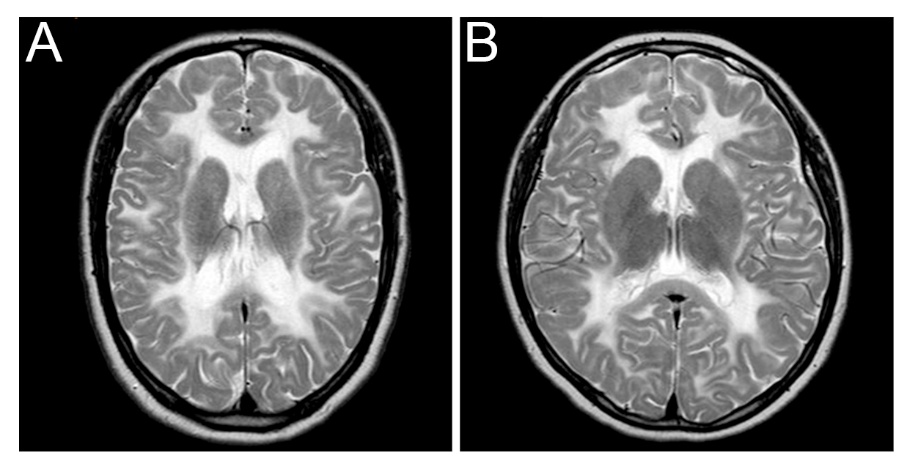


**Supplementary Figure 1. Magnetic resonance imaging of the asymptomatic sisters with white matter lesions.** Axial T2-weighted image of the asymptomatic sisters II.4 (A) and II.8 (B).
